# Unlocking the potential of established products: toward new incentives rewarding innovation in Europe

**DOI:** 10.1080/20016689.2017.1298190

**Published:** 2017-05-12

**Authors:** Gabrielle Nayroles, Sandrine Frybourg, Sylvie Gabriel, Åsa Kornfeld, Fernando Antoñanzas-Villar, Jaime Espín, Claudio Jommi, Nello Martini, Gérard de Pouvourville, Keith Tolley, Jürgen Wasem, Mondher Toumi

**Affiliations:** ^a^ Global Market Access and Pricing, IPSEN Pharma, Boulogne-Billancourt, France; ^b^ Pricing, Reimbursement and Market Access, Creativ-Ceutical, Paris, France; ^c^ Department of Economy and Business, University of La Rioja, Logroño, Spain; ^d^ Andalusian School of Public Health, Granada, Spain; ^e^ Department of Pharmaceutical Sciences, University of Eastern Piedmont, Novara, Italy; ^f^ Centre for Research on Health and Social Care Management (CERGAS), Bocconi University, Milan, Italy; ^g^ Drugs & Health, Rome, Italy; ^h^ Department of Economics, ESSEC Business School , Cergy-Pontoise, France; ^i^ Tolley Health Economics Ltd, Buxton, UK; ^j^ Department of Economics, University Duisburg-Essen, Essen, Germany; ^k^ Department of Public Health, Aix-Marseille University, Marseille, France

**Keywords:** Established products, extension of indication, new indication, repurposing, repositioning, pricing, reimbursement, life cycle management

## Abstract

**Background**: Many established products (EPs – marketed for eight years or more) are widely used off-label despite little evidence on benefit–risk ratio. This exposes patients to risks related to safety and lack of efficacy, and healthcare providers to liability. Introducing new indications for EPs may represent a high societal value; however, manufacturers rarely invest in R&D for EPs. The objective of this research was to describe incentives and disincentives for developing new indications for EPs in Europe and to investigate consequences of current policies.

**Methods**: Targeted literature search and expert panel meetings.

**Results**: Within the current European-level and national-level regulatory framework there are limited incentives for development of new indications with EPs. Extension of indication normally does not allow the price to be increased or maintained, the market protection period to be extended, or exclusion from a reference price system. New indication frequently triggers re-evaluation, resulting in price erosion, regardless of the level of added value with the new indication. In consequence, manufacturers are more prone to undertake R&D efforts at early to mid-stage of product life cycle rather than with EPs, or to invest in new chemical entities, even in therapeutic areas with broad off-label use. This represents a potentially missed opportunity as developing new indications for EPs offers an alternative to off-label use or lengthy and expensive R&D for new therapies, opens new opportunities for potentially cost-effective treatment alternatives, as well as greater equity in patients’ access to treatment options.

**Conclusion**: There are potential benefits from the development of new indications for EPs that are currently not being realized due to a lack of regulatory and pricing incentives in Europe. Incentives for orphan or paediatric drugs have proven to be effective in promoting R&D. Similarly, incentives to promote R&D in EPs should be developed, for the benefit of patients and healthcare systems.

## Introduction

Although investment in pharmaceutical research and development (R&D) has shown continual increase, productivity has been declining over the last decade.[1] Therapeutic innovation has become more challenging and pharmaceutical companies are looking for solutions to reduce the high attrition rate and offer patients better and safer drugs.[2] Re-purposing of established products (EPs), defined for the purpose of this study as marketed for eight years or more (as drugs benefit from eight years of data exclusivity after marketing authorization in the European Union), represents an opportunity for addressing this problem. Indeed, finding new uses for drugs that are already used for other conditions has great promise for rapidly bringing new treatments to patients, as has been recognized in a recent circular on the re-purposing of established medicines by the European Commission Expert Group on Safe and Timely Access to Medicines for Patients (STAMP).[3] As EPs have already cleared several key steps along the development path they represent safer molecules that can be used in high throughput screening platforms to discover new treatments for other disease. There is also considerable knowledge that has been gathered from long-term use of these drugs through off-label use, even though there is often little evidence on the benefit–risk ratio. However off-label use exposes patients to risks associated with safety and lack of efficacy, and healthcare providers to liability.[4,5]

Although introducing new indications for EPs could produce additional societal value at lower R&D cost, manufacturers may be reluctant to invest in R&D for EPs. We assume that they face significant financial disincentives, preferring to develop new molecular entities rather than unlocking the full therapeutic potential of EPs. Indeed, after costly development and regulatory costs, in many European jurisdictions EPs typically face price cuts, associated with generic competition, price negotiations or inclusion in reference price groups. Moreover, new indications – even if demonstrating a higher value of the drug compared to existing alternatives – often lead to price reductions in Europe due to a combination of price/volume agreements, external reference pricing or budget impact analysis.

The objective of this research was to identify and review the incentives and disincentives for developing new indications for EPs in Europe and to investigate the consequences of the current policies, drawing on case studies in key European markets.

## Materials and methods

In a first step, a search was conducted for existing incentives for developing new indications as well as pricing rules for EPs in Europe, with a special attention on the five largest European markets, i.e. France, Germany, the UK, Italy and Spain. A targeted literature search in Medline (via PubMed) and of internet resources was performed, including the European Medicines Agency (EMA) website (http://www.ema.europa.eu) and relevant government and healthcare authorities’ websites (medicine agencies, HTA agencies) for, respectively, European-level and country-level information. In addition, grey literature and available proprietary resources (internal database and reports) were searched.

In a second step, a targeted search was performed aiming to analyse the consequences of the current regulations. For that purpose, the French health technology assessment (HTA) agency website was searched, i.e. *Haute Autorité de Santé* – HAS (http://www.has-sante.fr/), in order to retrieve all Transparency Committee (TC) opinions during one year (published online in 2013) and indexed as concerning an ‘extension of indication’. An analysis was conducted to identify how many of these opinions referred to EPs. EPs were classified as all products which obtained their first marketing approval at least eight years before the year TC opinion was issued, regardless of a previously authorized dosage form and brand name. Combination products were excluded. In addition, the HAS website was screened in order to identify cases illustrating an impact of the current regulations on products’ lifecycle management strategy. Four products with a decrease in price associated with generic entry and/or approval of new indication were selected. Pricing databases were also searched, i.e. AMELI (for prices in France) and IHS (for prices in Italy and Spain) in order to analyse the price evolution of the selected products.

As a final step two expert panel meetings were organized aiming to review and complete the findings as well as further discuss the consequences of existing regulations and propose possible policy improvements. Seven experts in the field of drug market access participated in the meetings: one from France, one from Germany, one from UK, two from Italy and two from Spain (see Table S1 in supplementary files for the key opinion leaders profiles). The first meeting was held in Paris, in July 2015 and the second in Milan, in November 2015.

## Current regulations for established products

### EU-level incentives for development of established products

**Figure 1.**
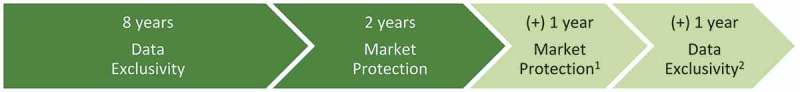
EU Provisions on extended data exclusivity and market protection periods. 1 – One additional year of market protection is granted if one or more new therapeutic indications bringing significant benefit in comparison with existing therapies are registered within the first eight years after initial approval. 2 – One stand-alone year of data exclusivity is granted for a new therapeutic indication for a well-established substance, provided that significant pre-clinical or clinical studies were carried out in relation to the new indication.

Under the European regulatory framework, marketing authorization holders can be granted one additional year of data exclusivity (Art. 10(5) Dir. 2001/83/EC) or one additional year of market protection (Art. 14(11) Reg. (EC) No 726/2004) for bringing a new indication into the market. Data exclusivity refers to the period of time during which a pharmaceutical company cannot cross-refer to the data in support of another marketing authorization (i.e. generics, hybrids and biosimilars cannot be validated by the EMA). An additional year of data exclusivity is granted for a new therapeutic indication for a well-established substance, provided that significant pre-clinical or clinical studies were carried out in relation to the new indication. Market protection refers to the period of time during which a generic, hybrid or biosimilar cannot be placed on the market, even if it has already received a marketing authorization. An additional year of market protection is granted for one or more new therapeutic indications which bring significant benefit in comparison with existing therapies. However, it applies exclusively to those medicinal products for which initial marketing authorization application was submitted since November 2005, and can be granted only if a new indication is registered within the first eight years after initial approval. Therefore, EPs cannot benefit from the provision of one additional year of market protection (Figure 1).

### Pricing rules affecting established products

EPs face price cuts in different European markets associated with a variety of reasons, including generic competition, inclusion in reference price groups, price negotiations with health care payers, or systematic price cuts after a period of marketing presence. Extension of indication implies a new HTA or value assessment and price re-negotiation (Table 1). The pricing policies and rules in each of the key European markets are reviewed in the following sections.

**Table 1.** Pricing rules for EPs in the five biggest European markets.[6,10,17–19]

Country	HTA submission at new indication	Price revision at new indication	Regular price revisions	Reference price groups	Price erosion at generics entry
France	✓	✓	✓	✓	YES, 20% price reduction of off-patent original at first generic entry and further 12.5% reduction after 18 months.
Italy	✓	✓	✓	✓	YES, price of the first generic must be 20% below the price of off-patent original, but the difference in prices is larger than 20%. This, together with generic reference pricing (and reference price sets at the minimum level) stimulates price erosion of off-patent original.
Spain	✓	✓	✓	✓	YES, price of the first generic must be 40% below the price of off-patent original; price of the off-patent original must decrease to the generic price, otherwise it is excluded from reimbursement.
Germany	na	na	✓	✓	YES, 10% mandatory discount on off-patent originals and generics plus 6% additional discount on non-reference priced off-patent originals and generics.
UK	✓	Possible evaluation by HTA bodies: NICE, SMC, AWMSG, followed by patient access scheme (typically a confidential price discount)			NO, free pricing but there is a strong competition due to very low prices of generics.

na: not applicable.

#### France

An extension of indication requires the manufacturer to provide a new submission to the TC, the French HTA agency (the law should be in force in 2016), and their assessment will result in price re-negotiation based on the outcome of the TC assessment. Regular HTA and price reviews are conducted every five years and may lead to price reductions in the case of a substantial increase in sales volumes even if supported by new indications. Price volume agreements are standard in France and a new indication may impact the already approved price volume agreement. A price review might be also triggered by an entry of a new competitor. The price of the off-patent original is reduced by 20% at the first generic entry and further by 12.5% after 18 months. Off-patent originals and generics, for which generic substitution rates are below fixed levels at set intervals of time after the first generic entry (i.e. if below 60% after 12 months, 65% after 18 months, 70% after 24 months or 80% after 36 months), are included into the active substance-based internal reference price system. Reimbursement price is set at the average of the prices of all generics in the group and patients are liable for any excess amount if they refuse the generic.[6]

#### Italy

An extension of indication involves submission of a new dossier and price re-negotiation with the Italian Medicines Agency (AIFA). The Agency may review (or the company itself may ask to review) reimbursement status and drug price every two years. Price negotiations can be triggered by an extension of indication, introduction of a new dose or by an entry of a cheaper comparative drug; price cuts are common in those situations. If following an extension of indication, the list price (official/published price) is not re-negotiated, a managed market entry contract (financial and/or outcome-based) may be applied to the new indication, and in consequence different effective prices are possible for different indications. Different contracts may apply to different indications (for instance in oncology) and drugs registries consider the price of a drug depending on the indication for which it is prescribed.[7] However, differential pricing per indication is usually set for oncology drugs for which outcomes are easy to measure.

The price of generic drugs must be 20% lower than the pre-patent expiry price of the original product, but generic prices are usually lower.[8] Off-patent originals and generics are included in the active substance-based internal reference price system (RPS) since 2001. Products included in the RPS, with the same molecule (off-patent molecule, with at least one generic product available in the market) and package are reimbursed at the lowest available final price. On average, prices of products included in RPS decreased by an additional 13% compared to other products, and each entry of a new generic was associated with a price drop of around 2.8%.[9] Patients are liable to cover any excess amount above the reference price:[10] €954 million (8.7% of the gross public expenditure for retail drugs) were paid by patients due to generic reference pricing in 2015.[11]

Price-cuts for EPs have been frequent in Italy. The most important one, approved in 2003, defined a maximum price per DDD (defined daily dosage) per ‘homogeneous’ therapeutic class (IV or sub-VI in Anatomical Therapeutic Chemical classification system) and delisted all products with a price per DDD over the maximum level. As a consequence, most companies were forced to lower price to avoid delisting.[8] AIFA has recently carried out a new general price-renegotiation on the grounds of persistent price-differentials within the same ‘homogeneous’ therapeutic class. Companies that refused the price-cut were asked to pay back the additional financial burden for the public payer. Products were fully delisted if the manufacturer did not accept the price-cut or pay-back.[12]

#### Spain

In Spain, an extension of indication requires a submission of a new dossier to the General Sub-directorate of Quality of Medicines and Medical Devices (SGCMPS). A drug price is likely to be cut thereafter, often due to the budget impact. Formal price reviews are not scheduled; however, pricing may be a subject of review after one year. In addition, drugs marketed for more than 10 years and out of the RPS are subject to price reviews. The Spanish RPS came into effect in December 2000 and was first applied to off-patent drugs with the same active ingredient.

There has been an incentive towards extension of indication with a law dated 2006 (*Ley de Garantías y Uso Racional de los Medicamentos y Productos Sanitarios* – Law on guarantees and rational use of medicinal products and health products).[13] This law granted one additional year of exclusivity for drugs with extension of indication with regards to inclusion in the RPS and to the applicable mandatory discount. This exception is still applicable today.

All products not included in the reference price system have been subject to mandatory discounts since June 2010 (*Real Decreto-ley 8/2010, de 20 de mayo, por el que se adoptan medidas extraordinarias para la reducción del déficit público* – Royal Decree-Law 8/2010, of 20 May, on extraordinary measures to reduce Government deficit approved in May 2010).[14] The total discount on innovative/patented drugs is 7.5% (the manufacturer supports 5% or 7.5% if the product is subject to direct distribution) or 4% if the product has an orphan indication (only direct distribution; manufacturer supports overall amount). Since August 2011, products marketed for more than 10 years for which there is no generic alternative on the market (and which as a result cannot be included in the RPS) are subject to a total discount of 15%; the manufacturer supports 10% or 15% if the product is subject to direct distribution. The mandatory discounts that apply in Spain are summarized in Table 2. This higher discount only applies at 11 years if a new indication has been approved (Real Decreto-ley 9/2011 – decree-law 9/2011).[15] This is not an incentive specific to innovation with EPs, as the additional year may be obtained for an indication extension in its first years. However, it is requested that the new indication was approved during these first 10 years.

**Table 2.** Mandatory drug discounts in Spain.

		Mandatory discounts as % of the public price (including VAT)					
		Retail distribution				Hospital drugs subject to direct distribution	
Law	Drugs category	Manufacturer	Wholesaler	Pharmacist	Total	Manufacturer	Total
Decree-law 8/2010 approved in May 2010	Innovative/patented drugs	5%	0.41%	2.09%	**7.5%**	7.5%	**7.5%**
	Innovative orphan drugs	na	na	na	**na**	4%	**4%**
Decree-law 9/2011 approved in August 2011	Older originals^1^ without generics	10%	0.82%	4.18%	**15%**	15%	**15%**
	Off-patent drugs in the RPS	0%	0%	0%	**0%**	0%	**0%**
	Off-patent drugs in RPS groups that are inactive (owing to patent disputes).	5%	0.41%	2.09%	**7.5%**	7.5%	**7.5%**

VAT: value-added tax; na: not applicable.

^1^ Retail and hospital products marketed for over 10 years (or 11 years if a new indication has been approved), for which there is no generic alternative on the market (and which as a result cannot be included in the reference price system). Drugs are exempt from this discount if they are still patented in all EU member states.

In January 2004, the Law of Cohesion and Quality of the National Healthcare System (*Ley de Cohesión y Calidad del Sistema Nacional de Salud* – from May 2003) [16] led to a fundamental reform of the RPS. Indeed, since then, reference price groups have been formed for drugs with the same active ingredient and ‘route of administration’ (e.g. all oral forms of the active ingredient are grouped together). Thus, the Spanish clusters include very different pharmaceutical formulations in the same group; for example, parenteral route of administration includes subcutaneous, intramuscular and infusion presentations, regardless of the frequency of administration. Only patented ‘innovative forms’ (i.e. those with a different route of administration that improves efficacy/safety/benefits or with the same route of administration but added therapeutic value) can be excluded from the reference price system for a period of five years only. Eligibility for exclusion is assessed by the Spanish Agency for Medicines and Health Products (AEMPS).

The price of the first generic must be 40% below that of the original brand. Since 2000, the reference price (reimbursed amount) was based on the simple arithmetic mean of the daily treatment cost of the three lowest priced drugs (DDDs). However, as of 1 November 2011 (decree-law 9/2011 [15]) the reference price level for each group is calculated based on the lowest daily treatment cost using DDDs. Patients co-payment above the reference price level is not allowed and only products at or below the reference price are reimbursed.[17]

#### Germany

In Germany, since the AMNOG law (*Arzneimittelmarkt-Neuordnungsgesetz*) became effective in January 2011, an extension of indication triggers a new assessment and a new price negotiation. This is valid however only for drugs which were launched in their first indication after 2010. Extensions of indication with EPs are therefore not subject to any assessment and the reimbursement for new indication is effective directly after market approval.

There is a 10% mandatory discount on prices of all off-patent originals and generics and 6% additional discount on those which are not included into the internal reference price system. Non-reference priced patented drugs are subject to a 7% mandatory discount.

Reference price groups can include all types of drugs (patented, off-patent and generics) considered to be therapeutically interchangeable. The reimbursement price is based on a weighted scale of the prices of drugs included in the group; it is calculated by a regression analysis (econometric model) and must be in the lowest third of the price range. Patients are liable for any excess over the reference price.[18]

#### UK

Manufacturers are free to set the drug price but there is a strong competition, leading to low prices of generics. The majority of generics are assigned to category M of Part VIIIA of the Drug Tariff list which means that reimbursement prices for these products are calculated based on volume-weighted actual average selling prices. Most prescriptions in the UK are made by an international non-proprietary name resulting in effective generic substitution and quick erosion of the sales of off-patent originals if they do not adjust the price to the generic price level.[19] In the UK, extensions of indication should be notified to horizon scanning agencies such as PharmaScan for potential HTA assessment (National Institute for Health and Care Excellence (NICE), All Wales Medicines Strategy Group (AWMSG), Scottish Medicine Consortium (SMC)). As part of the HTA review, a voluntary and undisclosed discount (net price decrease) might be offered by the manufacturer to improve its cost-effectiveness, or to manage the uncertainty related to a potential wider use of the drug. This may indeed support recommendation of the drug by HTA bodies such as NICE, SMC or AWMSG.

### Other regulations affecting established products

When applying for a market approval of a new indication within the EU, manufacturers may be required to submit a risk-management plan (RMP) to the EMA.[20] A RMP may be applied not only to the new indication but to the overall population. Setting up and executing RMPs represents burden for both manufacturers and physicians.

When developing a new indication for an authorized medicinal product protected either by a patent or a supplementary protection certificate, manufacturers also have to comply with the European Paediatric Regulation.[21] They have to submit a paediatric investigation plan (PIP) and unless they benefit from a waiver they will have to conduct expensive additional clinical trials in children.

## Consequences of current regulations

### Impact on the number of new indications with established products: the French case study

**Figure 2.**
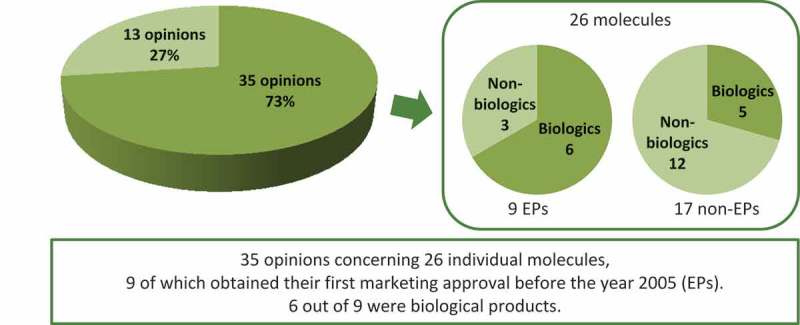
HAS opinions published on-line in 2013 and indexed as concerning an ‘extension of indication’.

In the year 2013, the French HTA Agency – HAS – published on its website 48 opinions concerning an ‘extension of indication’ for individual molecules.^1^ ^1^Out of the 48 opinions, 26 were issued in the year 2013, 21 in the year 2012 and one in the year 2011. Out of these 48 opinions, 13 concerned an extension of an existing indication to another age-group, mainly from an adult to paediatric population. The remaining 35 opinions referred to 26 individual molecules of which nine obtained their first marketing approval eight years before the year TC opinion was issued and thus were considered EPs (Figure 2). The majority of these (six out of nine) were biological products, i.e. adalimumab (Humira®, Abbvie), antilymphocyte globulins (Fresenius), infliximab (Remicade®, MSD), interferon beta-1a (Rebif®, Merck Serono), rituximab (Mabthera®, Roche) and trastuzumab (Herceptin®, Roche) (Table 3). While biologics represent 67% of the new indications with EPs, they represent only 29% of the new indications with non-EPs (See Table S2 in supplementary files for the description of the 17 non-EPs).

**Table 3.** EPs for which French HTA Agency published one or more opinions concerning an ‘extension of indication’ in 2013.

Molecule	Brand(s)	Manufacturer	First Marketing Approval^1^	Biological Product
Antilymphocyte globulins	x	Fresenius	6 May 1998	✓
Rituximab	Mabthera®	Roche	2 June 1998	✓
Telmisartan	Micardis®	Boehringer Ingelheim	16 December 1998	
	Pritor®	Bayer	11 December 1998	
Interferon beta-1a	Rebif®	Merck Serono	29 March 1999	✓
Infliximab	Remicade®	MSD	13 August 1999	✓
Trastuzumab	Herceptin®	Roche	28 August 2000	✓
Oxycodone	Oxycontin PR®	Mundipharma	5 December 2000	
	Oxynorm®	Mundipharma	11 June 2003	
Adalimumab	Humira®	Abbvie	8 September 2003	✓
Everolimus^2^	Certican®	Novartis	15 April 2004	

^1^ Regardless of a dosage form.

^2^ Everolimus is also marketed by Novartis under the brand name Afinitor® (date of marketing approval: 3 August 2009) and Votubia® (date of marketing approval: 2 September 2011); each brand is approved in different set of indications.

In the meantime, the French National Agency of Medicine and Health Products Safety introduced in December 2011 a framework for the potential use of one drug outside of the scope of the approved indication.[3] Temporary recommendation for use (RTU – *Recommandation Temporaire d’Utilisation*) allows off-label when there is an unmet therapeutic need and the benefit/risk ratio is assumed to be favourable based on the available data. Even if an RTU may be considered as a solution to balance off-label use, they are only granted for three years maximum and therefore are only a temporary solution for off-label use of EPs.

### Outcome of expert panel meetings and case studies

The board of experts reviewed current regulations and acknowledged that there is a likely loss of opportunity due to a lack of incentives, meaning many EPs are not further developed for new indications and patient populations. In the markets reviewed the incentive structure encourages R&D for the development of new molecules that will get patent protection and thus price protection.

The expert panel considered that serendipity plays an important role in the life of the product and that there is untapped potential with EPs. Indeed, current regulations are a source of loss of opportunity as prices are likely to decrease even when a new indication is approved. It was recognized that if an established product is bringing a clear therapeutic value for patients and society, then that innovation should be rewarded. Manufacturers should be incentivized to play an active role in clinical trials with EPs, instead of mostly relying on externally sponsored studies.[22]

**Figure 3.**
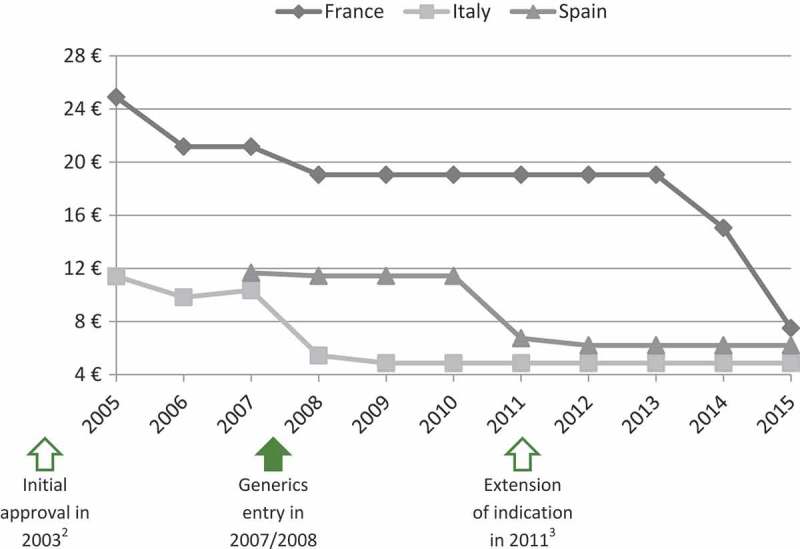
Price evolution: Ramipril (Tritace®/Triatec®, Sanofi-Aventis)^1^ price per 10 mg tablets; 28 or 30 units. 1 – Only generic forms available in Spain. 2 – Initial marketing approval in the treatment of hypertension and secondary prevention after acute myocardial infarction. 3 – Extension of indication to the treatment of renal disease and symptomatic heart failure. Source of prices: AMELI (France) and IHS (Italy, Spain) database.

**Figure 4.**
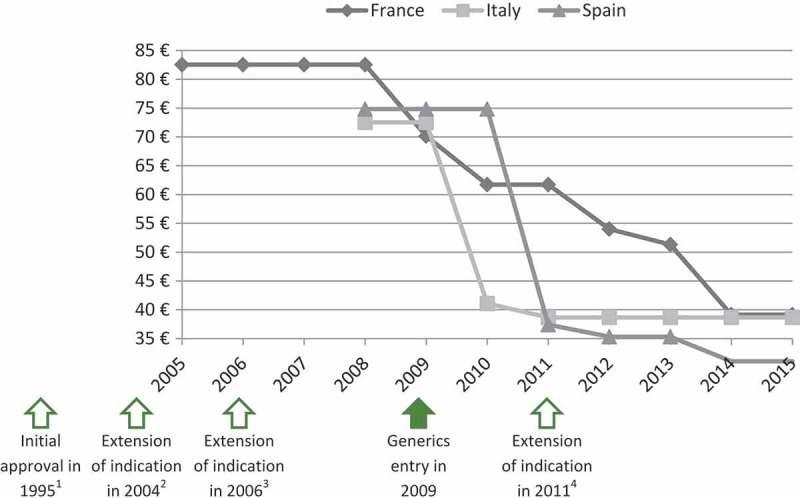
Price evolution: Valaciclovir (Zelitrex®/Valtrex®, GSK) price per 500 mg tablets; 42 units. 1 – Initial marketing approval in herpes simplex virus (HSV) infections. 2 – Extension of indication to varicella zoster virus (VZV) infections. 3 – Extension of indication to cytomegalovirus (CMV) infections. 4 – Extension of indication to VZV infections in immunocompromised patients. Source of prices: AMELI (France) and IHS (Italy, Spain) database.

The panel of experts selected four case studies among the EPs evaluated by the TC in 2011, 2012 and 2013 to illustrate that extension of indication does not permit to increase or maintain the price. As shown in Figures 3 and 4, prices of ramipril (Tritace®/Triatec®, Sanofi-Aventis) and valaciclovir (Zelitrex®/Valtrex®, GSK) decreased following generics entry and then continued to decrease, in spite of the subsequent introduction of new indications.

**Figure 5.**
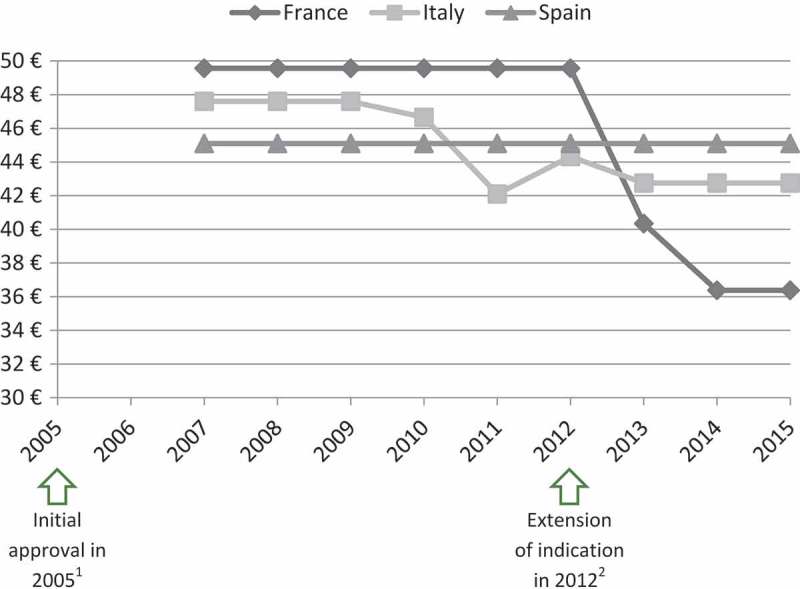
Price evolution: Ivabradine (Procoralan®, Servier) price per 5 mg tablets; 56 units. 1 – Initial marketing approval in chronic stable angina pectoris in coronary artery disease. 2 – Extension of indication in chronic New York Heart Association (NYHA) class II to IV heart failure with systolic dysfunction. Source of prices: AMELI (France) and IHS (Italy, Spain) database.

**Figure 6.**
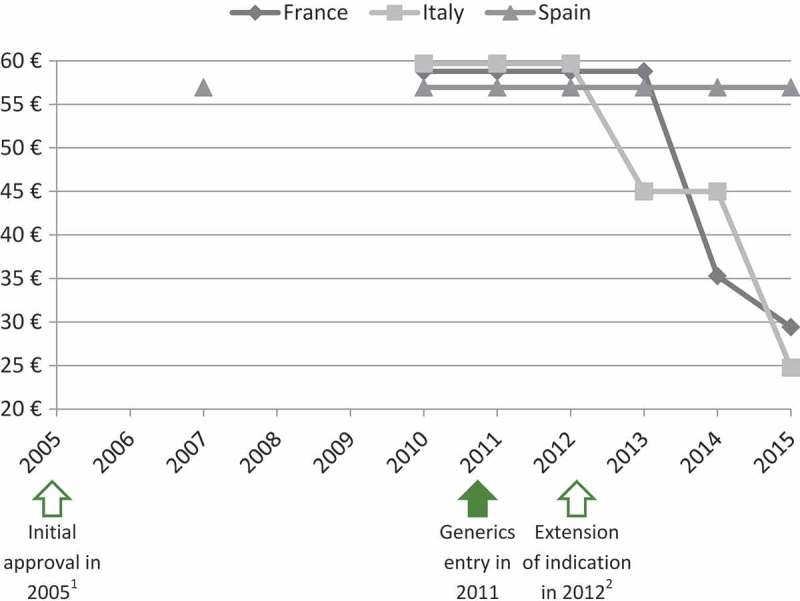
Price evolution: Eplerenone (Inspra®, Pfizer) price per 50 mg tablets; 30 units. 1 – Initial marketing approval in left ventricular dysfunction and clinical evidence of heart failure after recent myocardial infarction. 2 – Extension of indication in chronic New York Heart Association (NYHA) class II heart failure and left ventricular systolic dysfunction. Source of prices: AMELI (France) and IHS (Italy, Spain) database.

In the case of ivabradine (Procoralan®, Servier) and eplerenone (Inspra®, Pfizer) prices eroded, even though new indications were introduced before the loss of patent exclusivity (Figures 5 and 6).

The experts highlighted that eplerenone is prescribed for heart failure disease, which is an indication with a very large target population and a wide range of treatment options.[23,24] Price-volume agreements or budget-impact analysis could therefore explain the price decrease.

## Discussion

A key finding from the review is that there are clear disincentives for the development of new indications for EPs. These disincentives are mainly leading to price erosion, but also to additional, potentially burdensome, regulatory requirements such as development and implementation of RMPs and/or PIPs.

Within the current regulatory and the pricing rules framework there is a lack of clear incentives for development of new indications with EPs. Introducing an extension of indication normally does not allow the price to be increased or maintained, the market protection period to be extended or exclusion from a reference price group. Indeed, a new indication frequently triggers a price re-evaluation, often resulting in price cuts. As a consequence, manufacturers appear rarely to develop new indications for EPs, instead being prone to undertake R&D efforts either for new chemical entities or at early to mid-stage of a product’s life cycle rather than with EPs.[25] Due to a lack of incentives for developing new indications for EPs, valuable treatment options may therefore never be reaching the market for value assessment by HTA bodies with the potential to bring additional benefits to patients and society.

### Impact on lifecycle management strategy

In order to protect a price and revenue, and counterbalance additional R&D investments, new indications are more likely to be pursued during early to mid-stage of products life-cycle rather than for EPs, as illustrated with an example of bevacizumab (Avastin®, Roche). After its initial approval for metastatic colorectal cancer (mCRC) in January 2005, nine more indications were launched over the following seven-year timespan; four in the year 2007 (breast cancer, non-small cell lung cancer, renal cell cancer and a different treatment regimen for mCRC), one in 2009 (a different treatment regimen for breast cancer), three in 2011 (ovarian, fallopian tube or peritoneal cancer and two different treatment regimens for breast cancer) and one in 2012 (different stage of ovarian, fallopian tube or peritoneal cancer).[26] The work of Murteira et al. [25] revealed that such a strategy is commonly practised. The majority of repositioning cases^2^ ^2^Repositioning is defined as the process of finding a new therapeutic use for an already known drug. analysed in this study, i.e. 83.3% in France, 88.9% in Germany and 93.8% in the UK, were approved before patent expiry of the original product.[25] Our analysis of HAS reports of extension of indication also shows that biologics represent a high proportion of the EPs used for extension of indication (67% while only 29% of the non-EPs in this research were biologics), as they are normally less exposed to follow-on products (generics/biosimilars or hybrids) competition than non-biologics.

### Impact on off-label use

A lack of incentives to develop additional indications may result in wide off-label use of some EPs. Two types of situations may lead to this off-label use:

- Extension of use to populations or in conditions that are not included in clinical trials and therefore in product labelling. A drug is prescribed in the approved indication but it is used in a different patient population (paediatric, elderly, ethnic minorities or female population for instance), dosage, or dosage form.
- Extension of indication due to serendipity or an empirical finding. In this case a drug may be used in a quite distinct indication from the one for which it was initially labelled.

Systemic lupus erythematosus (SLE) – a chronic autoimmune disease with an estimated prevalence at 12.5–78.5 cases per 100,000 population in Europe and the USA [27] – is an example of a therapeutic area in which a wide range of drugs are used outside approved indications. Most of them are EPs with no regulatory driven development in this specific condition. However, in 2011 belimumab (Benlysta®, GSK) – a fully-humanized monoclonal antibody – became the first drug for 50 years to be licensed in SLE (Table 4).

**Table 4.** Drugs used in the treatment of SLE.

Therapeutic Class	Drug	SLE indication		First MA
		USA	EU	
NSAID	Aspirin	✓	x	1900s
Corticosteroids	Prednisolone	✓	✓^1^	1950s
	Hydrocortisone	✓	✓^1^	1950s
Monoclonal antibodies	Belimumab	✓	✓	2011
	Rituximab	x	x	1997
Immunosuppressants	Mycophenolate mofetil	x	x	1995
	Azathioprine	x	✓^1^	1990s
	Abatacept	x	x	2007
	Tacrolimus	x	x	1994
	Thalidomide	x	x	1950s
	Cyclosporin	x	x	1983
Antimalarials	Hydroxychloroquine	✓	✓^1^	1950s
Cytotoxics	Methotrexate	x	x	1950s
	Cyclophosphamide	x	✓^1^	1959

^1^ Drugs having indications for SLE within non-centralized procedures in some EU countries.

MA – marketing approval, NSAID – nonsteroidal anti-inflammatory drugs, SLE – systemic lupus erythematosus

In Europe, an annual cost of treatment with old drugs ranges from approximately €70 to €2500 (based on ex-factory prices), i.e. an annual cost of hydroxychloroquine is €74, oral prednisolone €119, azathioprine from €83 to €250, cyclophosphamide from €266 to €532 and injectable hydrocortisone from €512 to €2559. In contrast, an annual cost of belimumab (assuming 15 administrations a year) can reach €14,680, which is six times more than the highest possible dose of injectable hydrocortisone and nearly 200 times more than hydroxychloroquine (data for the UK).[28] Four new molecular entities and one drug, already approved in another indication, are currently at the advanced stage of clinical development for SLE.[29] All of them are biological products and they are expected to be associated with high prices as compared with EPs used off-label in SLE (Table 5).

**Table 5.** New drugs in development for SLE.

Molecule (brand)	Manufacturer	Drug type	Phase of development	Status
Rigerimod (Lupuzor^TM^)	Immupharma	Oligopeptide	III	New drug
Atacicept	Merck Serono	Recombinant fusion protein	II/III	New drug
Blisibimod (A623)	Amgen	Recombinant fusion protein	III	New drug
Epratuzumab	UCB	Monoclonal antibody	III	New drug
Abatacept (Orencia®)^1^	BMS	Immunoglobulin fusion protein	III	Approved in other indication in 2007

^1^ Developed for lupus nephritis

**Figure 7.**
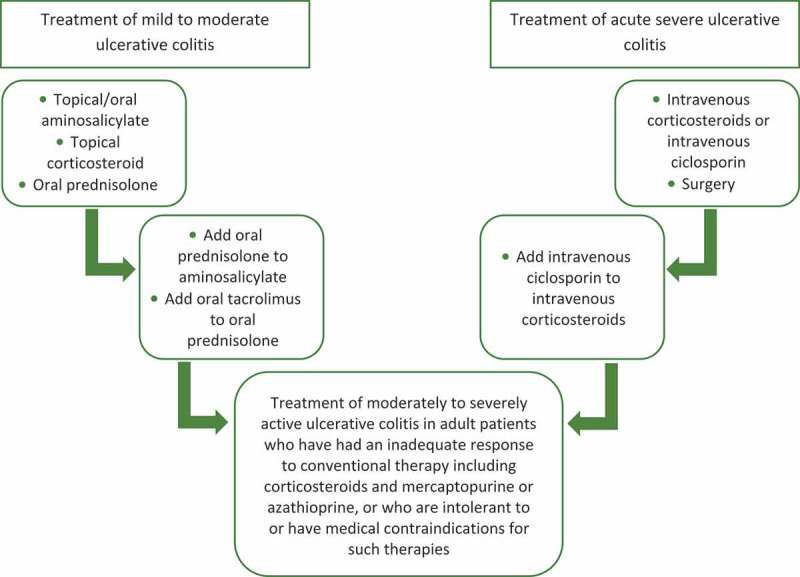
NICE guidelines for treatment of ulcerative colitis [30,31].

On the other hand when EPs are approved for a new indication they are likely to be considered in first-line before expensive biologics are prescribed. This is the case for bowel inflammatory disorders; anti-TNF are recommended in the UK as a rescue therapy after failure of the conventional therapy including aminosalicylate, corticosteroids and immunosuppressants (Figure 7).[30,31] EPs in this case provide a more affordable treatment option.

### Societal value of new indications with established products

The development of new indications for EPs can be a win-win solution for both patients and payers. It offers an alternative to off-label use of drugs, which is estimated to account for 15–20% of all prescriptions in general (data for France) [32] and 50%, or even more, in oncology.[33] Up to 81% of HIV patients receive at least one off-label prescription during their treatment.[34] Off-label use may pose a safety risk, if side-effects or interaction profile are not well documented for off-label indications. It also generates risks of additional costs without a proof of efficacy and exposes healthcare professionals to liability. Moreover, off-label prescription is often limited to patients treated by key opinion leaders, dosage forms are frequently not adapted to non-registered uses and, in some countries, drugs may not be reimbursed if administered outside approved indications.[33] It is suggested that a patient group that can be treated with a licensed drug will be treated better and in a more homogeneous way in comparison with a group where only off-label drugs are available.[35] New indications with EPs could therefore allow reducing the risks and lack of equity associated with off-label prescription.

The development of EPs offers, in addition, a faster option compared to lengthy and expensive development of new therapies. It has been estimated that *de novo* drug discovery and development lasts from 10 to 17 years and is associated with <10% probability of success, while in the case of repurposed drugs, the overall process may be shortened to 3–12 years. Repositioned drugs often move directly to phase II clinical trials, potentially reducing drug development time by 5–6 years.[36] Additionally, as safety accounts for almost 30% of drug failures in clinical trials,[37] EPs – for which large amounts of valuable real-life data on safety are available – should be considered as an interesting option to invest in. For a given drug, most side effects are not disease specific but mainly related to the mechanism of action. Therefore, the use of real-life data can help to substantially reduce the risk of failure when developing a new indication.

Despite the lack of regulatory incentives, several examples indicate that when it is possible to obtain a return from investment, manufacturers still manage to develop new indications for EPs demonstrating a high added value. One example is dimethyl fumarate which has been available in European markets for the treatment of psoriasis and as a food supplement for about 20 years. Biogen developed it under the brand name Tecfidera® for multiple sclerosis (MS). EMA Committee for Medicinal Products for Human Use (CHMP) initially denied granting Tecfidera® a new active substance (NAS) status. However, after being approved in the USA, Canada and Australia, under a threat of delaying the launch in Europe, CHMP decided to consider Tecfidera® as an NAS in 2013.[38] The drug, with its ability to reduce MS relapses by approximately 50%,[39] already benefited a large number of patients. As a further example Novartis developed and commercialized everolimus for three distinct indications under three different brand names – Certican® (2004), Afinitor® (2009) and Votubia® (2011) – and achieved differential pricing for individual indications. Two initially introduced brands are available in distinct doses: Certican®, approved for cancer therapy, is marketed as 0.25 mg, 0.5 mg and 0.75 mg tablets whilst Afinitor®, used in transplantology, is marketed as 2.5 mg tablets. Votubia®, despite being available similarly to Afinitor® as 2.5 mg tablets, was developed for use in a rare condition – tuberous sclerosis – and therefore, due to its orphan designation, benefits from 10 years of market exclusivity. In France, the cost per one day of treatment with Certican® is approximately €12. In case of Afinitor® and Votubia®, it is significantly higher and reaches €110 and €127, respectively.[40–43] A similar situation is observed in other European countries. 43] A similar situation is observed in other European countries. Pierre Fabre Dermatologie developed Hemangiol® – the first and only drug approved for the treatment of proliferating infantile haemangioma. Hemangiol® contains propranolol, a beta-blocker initially approved in the year 1980 and used for a number of indications such as hypertension, angina, arrhythmias, tachycardia, prophylaxis after myocardial infarction, prophylaxis of migraine, anxiety and other. As opposed to ‘old’ formulations of propranolol (tablets, modified-release capsules, solutions for injection, oral solutions), Hemangiol® is an oral solution specifically developed to suit the needs of infants (adapted unit dose, no harmful excipients and acceptable flavour). It has been positively received by payers and the drug achieved a significantly higher pricing as compared with other propranolol products.[28,43,44] However, these examples are rather an exception than the rule and require differentiated products, which is not always possible.

### Some incentives in place

The introduction of European level incentives for development of orphan and paediatric drugs resulted in an increased number of new approvals benefiting a range of previously disadvantaged patient groups.[45] Since 1999 orphan drugs developers have had protocol assistance, access to centralized authorization procedure with a possibility to access conditional approval on a case-by-case basis, 10 years of market exclusivity which may be increased by two years if a paediatric investigation plan is submitted and agreed, as well as reduced fees for regulatory activities.[46] Manufacturer of paediatric products, since 2007, can benefit from six months extension of patent protection for drugs authorized across the EU with the results of PIP studies included in the product information as well as from scientific advice and protocol assistance for questions relating to the development of drugs for children. Moreover, drugs developed specifically for paediatric use that are already authorized but not protected by a patent or supplementary protection certificate, can apply for a paediatric-use marketing authorization, i.e. PUMA. If PUMA is granted, the product will be granted 10 years of market protection.[47] Therefore there are no obvious legal obstacles for the introduction of incentives stimulating development of new indications for EPs with added value for patients and for society, which could be of a significant benefit for all healthcare system stakeholders.

Drug repositioning can be protected from a legal point of view through ‘new use patent’, also called ‘second medical use patent’. For example, the Swiss-form claim has been used until 2010 ‘Use of substance X in the manufacture of a medicament for the treatment of condition Y’.[48] The European Patent Convention 2000 (EPC 2000) introduced a new claim: ‘Substance X for use in the treatment of condition Y’ for the use of the same substance for a new condition.[49] To obtain such a patent claim, specific conditions should be met:

- The new use must be supported by evidence.
- Novelty and inventiveness over the previous use have to be established.

Therefore this type of patent cannot be claimed for EPs with known off-label use. Hence, even if such a patent can be claimed, it is often complicated for the manufacturer to enforce this patent and protect the new market. If a generic is already marketed for the first indication, it is very difficult to prevent prescription/delivery of the generic for the new indication. For example, pregabalin has been approved since 2004 for the treatment of generalized anxiety disorder and epilepsy. This patent expired in May 2013 and the data exclusivity period expired in July 2014. Pfizer was later awarded a Swiss-form claim for use of pregabalin for neuropathic pain and the patent is valid until July 2017.[50] In the UK, physicians usually prescribe by active ingredient (international non-proprietary name – INN) rather than a brand name. As a consequence, instead of delivering the brand name Lyrica®, pharmacists often deliver a generic for the pain indication. Pfizer sued the generic manufacturer for patent infringement. The first ruling ordered the National Health Services England to issue guidance recommending the prescription of the brand name for the pain indication and to require pharmacists to deliver Lyrica® for this indication.[50] The High Court ruled in a second instance that Pfizer’s claims were invalid and as such that there was no infringement. The judge however made the suggestion that the drug could be prescribed by its generic name for off-patent uses and by brand name for patented new indication. In that case, Pfizer claimed it had conducted more than 50 clinical studies to develop this new indication, involving some 12,000 patients.[51] This case highlights further the need to develop some type of protection for new indications with EPs.

### Proposition of incentives for the development of new indications with established products

**Figure 8.**
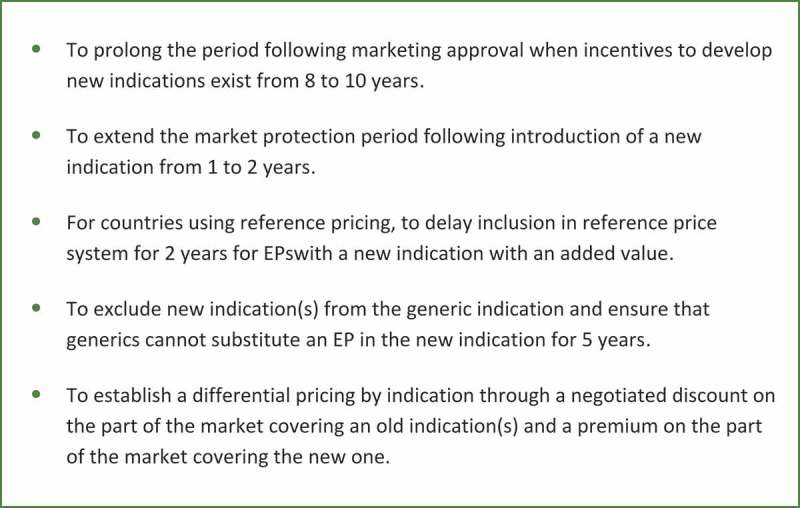
Proposition of incentives for the development of new indications with EPs.

In order to encourage R&D for EPs the following changes within the current regulatory and pricing regulations have been suggested by the authors (Figure 8):

- To prolong the period following marketing approval when incentives to develop new indications exist from eight to 10 years, i.e. additional time of market protection is granted providing a new indication is registered within the first 10 years after initial approval (instead of within the first eight years, like today).
- To extend the market protection period following introduction of a new indication from one to two years.
- For countries using reference pricing, to delay inclusion in reference price systems for two years for Eps with a new indication with an added value.
- To exclude new indication(s) from the generic indication and ensure that generics cannot substitute an EP in the new indication for five years.
- To establish a differential pricing by indication through a negotiated discount on the part of the market covering an old indication(s) and a premium on the part of the market covering the new one; this approach has been already adopted in Italy, but it is not practiced in other European countries. It requires that drug registries are implemented to identify the indication the drugs are prescribed for.

### Limitation and further areas of research

The board of experts pointed out that the value of a new indication should be established versus a new indication with no value for society. Therefore, in order to enable manufacturers to be eligible to potential incentives in new policy, it seems critical to consider the following issues when assessing a new indication for an established product:

1. Define which type of extension of indication represents a truly new indication.
2. Set criteria for evaluation of the added value of a new indication in order to measure the benefit of EPs for patients and for society.
3. Define segmentation criteria: consider the size of the new indication in comparison to the first indication.
4. Measure the loss of opportunity for society with the current regulatory and pricing policies.
5. Decide whether specific HTA based incentives also need to be created beyond regulatory incentives to encourage market access for re-purposed EPs.

These topics will be addressed through further research and expert panel discussions. The current rules for authorization of off-label shall also be further analysed as these rules might solve some problems linked to off-label use (notably liability of clinicians).

## Conclusion

There appears to be a high unmet need concerning the potential opportunity offered by the development of new indications for EPs, due to a lack of current regulatory and pricing rule incentives in Europe. Incentives for orphan drugs or paediatric indications have proven to be effective in promoting R&D. Similarly, greater incentives to promote R&D in EPs should be developed, for the expected benefit of patients and healthcare systems. Further research is needed to identify viable incentives toward late extension of indication in non-European countries, national regulatory frameworks for off-label prescriptions, previous suggestions to reduce the risks attached with off-label, and economic modelling of incremental innovation impact.

## Supplementary Material

Supplementary Material
