# Supplementary material for: Unlocking the potential of established products: toward new incentives rewarding innovation in Europe
Source: J Mark Access Health Policy. 2017 May 12;5(1):1298190. doi: 10.1080/20016689.2017.1298190 (PMC5508393; doi:10.1080/20016689.2017.1298190)
Supplement: Supplementary Material [file zjma_a_1298190_sm1542.docx]

Supplementary files

Table S1. Profiles of key opinion leaders participating in the expert panel meetings

| **Name** | **Country** | **Profile** |
| --- | --- | --- |
| Fernando Antoñanzas-Villar | Spain | Professor of Applied Economics at the University of La Rioja (Spain); former President of the Spanish Health Economics Association; member of the EU Health Scientific Panel for the Framework Program Horizon 2020 of the General Direction of Research and Innovation. |
| Jaime Espín | Spain | Professor at the Andalusian School of Public Health in Granada (Spain); former advisor to the European Commission during the High Level Pharmaceutical Forum (Working Group on Pricing); former member of the Experts Committee on Pharmaceutical Policies of the Pan American Health Organization (PAHO/WHO), and member of the panel of experts that worked on the WHO Guideline on Pharmaceutical Pricing Policies. |
| Claudio Jommi | Italy | Associate Professor of Management at the University of Eastern Piedmont, Department of Pharmaceutical Sciences, Novara (Italy); Scientific Director of the Pharmaceutical Observatory at the Centre for Research on Health and Social Care Management (CERGAS), Bocconi University (Italy); President of the Italian Health Economics Association. |
| Nello Martini | Italy | Director of Drugs & Health, Rome (Italy); former General Director of Italian Medicines Agency (AIFA); former Director of Hospital Pharmacy at Borgo Roma Polyclinic, Verona (Italy). |
| Gérard de Pouvourville | France | Chair Professor of Health Economics at the Paris ESSEC Business School (France) and member of the European Quality of Life Group (EuroQoL); former Chairman of the French Health Economists College and vice-president of the National Observatory for the Prescription and Usage of Drugs; scientific advisor to the French Ministry of Health on hospital funding systems and reforms for the past 20 years. |
| Keith Tolley | UK | Director of Tolley Health Economics, Buxton (UK); assessor for Scottish Medicines Consortium (SMC), All Wales Medicines Strategy Group (AWMSG) and previously consulted for the National Institute for Health and Care Excellence (NICE) Early Scientific Advice Programme. |
| Mondher Toumi | France | Professor of Public Health at Aix-Marseille University (France), visiting Professor at Beijing University (China), and Director of Creativ-Ceutical; in 2009 appointed as Director of the Chair of Public Health and Market Access at the University of Lyon 1; creator of the European Market Access University Diploma (EMAUD). |
| Jürgen Wasem | Germany | Professor of Health Economics in the Institute for Health Care Services Management and Research at the University Duisburg-Essen (Germany), President of the German Health Economics Association (DGGOE), member of the Board of Directors of Federal Association for Managed Care (BMC), Chair of Academic Advisory Board for the Further Development of Risk Adjustment at the Federal Insurance Office, Chairman of the Federal Arbitration Committee between Social Health Insurance Funds and the Federal Association of Self Employed Physicians (EBA), Chair of the Arbitration Committee and a member of the Scientific Committee of the International Health Economics Association (IHEA). |

Table S2. Non-EPs for which French HTA Agency published one or more opinions concerning an ‘extension of indication’ in the year 2013

| Molecule | Brand(s) | Manufacturer | First Marketing Approval^1^ | Biological Product |
| --- | --- | --- | --- | --- |
| Bevacizumab | Avastin® | Roche | 12 Jan 2005 | 🗸 |
| Apixaban | Eliquis® | BMS | 18 May 2011 |  |
| Vildagliptin | Galvus® | Novartis | 26 Sep 2007 |  |
| Eplerenone | Inspra® | Pfizer | 05 Jan 2005 |  |
| Sitagliptin | Januvia®  Xelevia® | MSD  Pierre Fabre | 21 Mar 2007 |  |
| Ranibizumab | Lucentis® | Genentech | 22 Jan 2007 | 🗸 |
| Nepafenac | Nevanac® | Alcon | 11 Dec 2007 |  |
| Saxagliptin | Onglyza® | BMS | 22 Nov 2011 |  |
| Abatacept | Orencia® | BMS | 21 May 2007 | 🗸 |
| Dexamethasone | Ozurdex® | Allergan | 27 Jul 2010 |  |
| Dabigatran | Pradaxa® | Boehringer Ingelheim | 18 Mar 2008 |  |
| Ivabradine | Procoralan® | Servier | 25 Oct 2005 |  |
| Eculizumab | Soliris® | Alexion | 21 Jun 2007 | 🗸 |
| Panitumumab | Vectibix® | Amgen | 03 Dec 2007 | 🗸 |
| Pazopanib | Votrient® | Glaxosmithkline | 14 Jun 2010 |  |
| Rivaroxaban | Xarelto® | Bayer | 09 Dec 2011 |  |
| Abiraterone | Zytiga® | Janssen-Cilag | 05 Sep 2011 |  |

1 – Regardless of a dosage form
